# The MHC locus and genetic susceptibility to autoimmune and infectious diseases

**DOI:** 10.1186/s13059-017-1207-1

**Published:** 2017-04-27

**Authors:** Vasiliki Matzaraki, Vinod Kumar, Cisca Wijmenga, Alexandra Zhernakova

**Affiliations:** 1 Department of Genetics, University of Groningen, University Medical Center Groningen, PO Box 30001, 9700 RB Groningen, The Netherlands; 2 Department of Immunology, KG Jebsen Coeliac Disease Research Centre, University of Oslo, PO Box 4950 Nydalen, 0424 Oslo, Norway

## Abstract

In the past 50 years, variants in the major histocompatibility complex (MHC) locus, also known as the human leukocyte antigen (HLA), have been reported as major risk factors for complex diseases. Recent advances, including large genetic screens, imputation, and analyses of non-additive and epistatic effects, have contributed to a better understanding of the shared and specific roles of MHC variants in different diseases. We review these advances and discuss the relationships between MHC variants involved in autoimmune and infectious diseases. Further work in this area will help to distinguish between alternative hypotheses for the role of pathogens in autoimmune disease development.

**Electronic supplementary material:**

The online version of this article (doi:10.1186/s13059-017-1207-1) contains supplementary material, which is available to authorized users.

## Introduction

The major histocompatibility complex (MHC) locus, also known as the human leukocyte antigen (HLA) locus, spans around 4 Mbp on the short arm of chromosome 6 (6p21.3; Box 1). Molecules encoded by this region are involved in antigen presentation, inflammation regulation, the complement system, and the innate and adaptive immune responses, indicating the MHC’s importance in immune-mediated, autoimmune, and infectious diseases [1]. Over the past 50 years, polymorphisms in the MHC locus have been shown to influence many critical biological traits and individuals’ susceptibility to complex, autoimmune, and infectious diseases (Boxes 2 and 3). In addition to autoimmune and inflammatory diseases, the MHC has recently been found to play a role in some neurological disorders [2–6], implicating autoimmune components in these diseases.

The genetic structure of the MHC is characterized by high levels of linkage disequilibrium (LD) compared to the rest of the genome, which means there are technical challenges in identifying MHC single nucleotide polymorphisms (SNPs), alleles, and amino acids. However, the recent availability of dense genotyping platforms, such as the custom-made Illumina Infinium SNP chip (Immunochip) [7], and of MHC reference panels has helped to fine-map the locus, improving our understanding of its disease associations and our ability to identify functional variants.

In this review, we discuss recent advances in mapping susceptibility variants in the MHC, using autoimmune and infectious diseases as examples (Boxes 2 and 3). We also discuss the relationships between the MHC variants involved in both autoimmune and infectious diseases and offer insights into the MHC-associated immune responses underlying disease onset and pathogenesis. Finally, we discuss future directions for studying genetic variation in the MHC and how learning about the variation at this locus will aid understanding of disease pathogenesis.

## Advances in mapping susceptibility variants in the MHC locus

Several computational and empirical challenges complicate the mapping of MHC susceptibility variants. One fundamental challenge is that the MHC has many sequence and structural variations [8], which differ between populations and complicate haplotype inference. Another is that high and extensive LD in the locus makes it difficult to identify causal and independent loci. Non-additive allelic effects in the MHC, and epistatic effects between the MHC and other loci, can also complicate inference of the underlying haplotype structure and disease susceptibility variants.

In recent years, large volumes of sequencing data have made it possible to impute MHC variation on a wide scale, thereby improving our understanding of variability at this locus and of the haplotype structures and enabling reference panels to be created. The availability of accurate reference panels and a large number of genotyped individuals has allowed the identification of independent variants and improved our understanding of their contribution to disease heritability and pathways underlying disease biology [9, 10].

### Advances in laboratory-based mapping of MHC variation

Increased throughput, accuracy, and read length in next-generation sequencing (NGS) technologies, as well as the development of user-friendly bioinformatics tools, have enabled higher resolution MHC typing [11]. For instance, whole-genome sequencing (WGS) was successfully used to type HLA-A alleles at full resolution in 1070 healthy Japanese individuals [12] and to fully evaluate HLA-E variability in West African populations [13]. However, the main problem with MHC sequencing using current technologies is the relatively short read lengths, which limit the amount of allelic data that can be generated at a high resolution. Long-range PCR amplification approaches, such as the use of PacBio systems for single molecule real-time sequencing, significantly increase read-length and the resolution for typing MHC alleles [14]. In a comparison of MHC typing in an Indian population using sequence-specific primers, NGS (Roche/454) and single molecule sequencing (PacBio RS II) platforms, higher resolution typing was achieved for MHC class I (HLA-A, HLA-B, and HLA-C ) and class II genes (HLA-DRB1 and HLA-DQB1) using the PacBio platform, with a median read length of 2780 nucleotides [15].

**Fig. 1:**
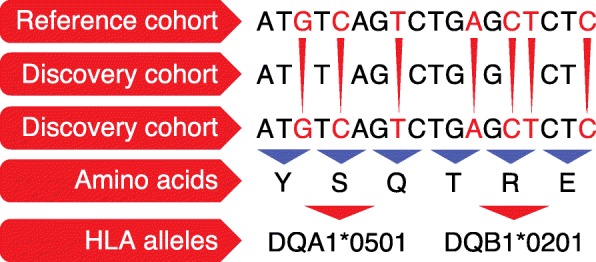
Major histocompatibility complex imputation. A reference cohort of subjects for whom both genetic information and classic human leukocyte antigen (HLA) typing is available can be used to infer the missing (untyped) genotypes and amino acids in a discovery cohort. This allows imputed variants to be tested for their associations with a disease of interest. The figure shows imputation points to classic alleles associated with celiac disease risk in the MHC region on chromosome 6. *Y* tyrosine, *S* serine, *Q* glutamine, *T* threonine, *R* arginine, *E* glutamic acid

High-density SNP panels, such as the Immunochip platform [7], which has been widely implemented in immunogenetics studies, are a cheaper, faster, and easier alternative to genotyping than direct MHC typing and NGS methods. The Immunochip contains a dense panel of SNPs from the MHC locus, which enables missing classic MHC variants to be inferred in silico, where the imputation is based on the haplotype structure present in large reference panels (Fig. 1). This fine-mapping approach has been used for several autoimmune and inflammatory diseases (Table 1) and for a few infectious diseases (Additional file 1), thereby allowing comprehensive interrogation of the MHC. Moreover, population-specific reference panels made by deep sequencing and used to impute genotypes allow identification of very rare variants and novel single-nucleotide variants in the human genome. This is illustrated by a recent study in which the authors first built a Han Chinese MHC-specific database by deep sequencing the region in 9946 patients with psoriasis and 10,689 healthy controls, and then used this reference panel to impute genotype data to fine-map psoriasis-associated variants [16]. Notably, functional variants in non-coding regions can be identified, as shown in a Japanese cohort of 1070 healthy individuals [12]. These variants would be impossible to discover using SNP microarrays or low coverage sequencing on the same sample size (Fig. 1, Table 1).

**Table 1:** Major histocompatibility complex (MHC) associations to autoimmune diseases, as described by fine-mapping studies

Disease	Classic associated locus	Fine-mapping: top locus	HLA class for top association	Fine-mapping: independent associations at *P* < 5 × 10^–8^	HLA class for independent association	Population	Sample size	Reference
RA	Shared epitope (HLA-DRB1 AA70-74)	HLA-DRB1: AA 11, 13, 71, 74	II	HLA-B AA 9	I	European	5018 individuals with seropositive RA, 14,974 unaffected controls	[19]
				HLA-DPB1 AA 9	II			
		HLA-DRB1: AA 11, 57, 74	II			Chinese, Korean	2782 seropositive RA cases, 4315 controls	[34]
		HLA-DRB1: AA 11, 71, 74	II	HLA-DPB1 AA 84	II	Japanese	6244 cases, 23,731 controls	[25]
				HLA-DOA (rs378352)	II			
				HLA-B 40:02	I			
CeD	HLA DQ locus (DQ2, DQ8)	HLA-DQA1: AA 25 and 47; HLA-DQB1: AA 57 and 74	II	AA9 in HLA-DPβ1	II	European	12,016 cases, 11,920 controls	[35]
				rs2301226 (near HLA-DPB1 gene)	II			
				HLA-B*08:01	I			
				HLA-B*39:06	I			
				rs1611710 (HLA-F)	I			
Ps	HLA-Cw6 (HLA-C 0602)	HLA-C*06:02	I	HLA-C*12:03	I	European	9247 patients, 13,589 controls	[36]
				HLA-B amino acid 67	I			
				HLA-B amino acid 9	I			
				HLA-A amino acid position 95	I			
				HLA-DQα1 amino acid position 53	II			
AS	HLA-B27	HLA-B*27:02 and B*27:05	I	HLA-A*02:01	I	European	9069 AS cases, 13,578 controls	[37]
				HLA-DPB1 (rs1126513)	II			
				HLA-DRB1*01:03	II			
				other HLA-B alleles (B*07:02) (protective), B*13:02, B*40:01, B*40:02, B*47:01, B*51:01 and B*57:01 (protective)	I			
SLE	HLA-DRB1 (*03:01 and *15:01); TNF-308G/A; C4A null allele	HLA-DRB1 AA 11, 12, 26	II	HLA-DQB1*02	II	Korean	849 SLE cases, 4493 controls	[38]
		HLA-DRB1*15:01-HLA-DQB1*06:02	II					
		DRB1*0301	II	rs419788 in intron 6 of the SKIV2L	III	European	314 trios	[39]
		rs1150753 in intronic region of TNXB	III	HLA-DRB1*03:01	II	European	3701 cases, 12,110 controls	[40]
				HLA-DRB1*08:01	II			
				HLA-DQA1*01:02	II			
				rs8192591 (upstream of NOTCH4)	III			
				rs2246618 near MICB	I			
		HLA-DRB1 AA 13, 11; HLA-DRB1*15:01-HLA-DQB1*06:02	II			6 Asian cohorts (from China, Korea, Japan)	4478 cases, 12,656 controls	[41]
T1D	DRB1, DQB1, in particular heterozygosity for HLA-DR3/HLA-DR4	HLA-DQβ1 position 57; HLA-DRβ1 positions 13; HLA-DRβ1 positions 71	II	HLA-B*39:06 HLA-B*18:01 HLA-B*50:01 HLA-DPB1*04:02 HLA-DPB1*01:01 HLA-A AA62 HLA-A*03 HLA-A*24:02	I I I II II I I I	European	8095 cases, 10,737 controls	[43]
		DRB1, DQB1	II	HLA-B*39 HLA-B*13 HLA-B*50 HLA-B*18 HLA-B*38 (protective) HLA-A*24	I I I I I I	European	2300 families	[42]
MS	HLA-DRB1*15:01	HLA-DRB1*15:01	II	DRB1 alleles: *03:01, *13:03, *04:04, *04:01, and *14:01	II	European	5091 cases, 9595 controls	[45]
				HLA-A*02:01	I			
				AA 65 of HLA-DPβ1	II			
				rs2516489 in MICB-LST1 locus	III			
				HLA-B*37	I			
		HLA-DRB1*15:01	II	HLA-DRB1*03:01, *13:03, *08:01	II	European	17,465 cases, 30,385 controls	[44]
				HLA-DQB1*0302	II			
				HLA-A*02:01 (protective)	I			
				HLA-B*44:02, *38:01, *55:01 (protective)	I			
CD	HLA class II and HLA-C	HLA-DRB1*01:03	II	HLA-C*06:02	I	European	18,405 cases, 34,241 controls	[46]
UC	HLA class II	rs6927022(HLA-DQA1); HLA-DRB1*01:03	II	HLA-C*12:02	I	European	14,308 cases, 34,241 controls	
Graves’ disease	DRB1*03:01-DQA1*05:01-DQB1*02:01 (DR3-DQ2 haplotype), HLA-C	HLA-DPB1 AA35, AA55, DPB1*05:01	II	HLA-DPβ1 AA9	II	Japanese	1956 cases, 7047 controls	[24]
				HLA-A AA9	I			
				HLA-B AA45, AA67	I			
DM	HLA-DPB1	HLA-DPB1*17	II	HLA-DBP1 (rs7750458-A)	II	Han Chinese	127 cases, 1566 controls	[47]

*RA* rheumatoid arthritis, *CeD* celiac disease, *Ps* psoriasis, *AS* ankylosing spondylitis, *SLE* systemic lupus erythematosus, *T1D* type 1 diabetes, *MS* multiple sclerosis, *CD* Crohn’s disease, *UC* ulcerative colitis, *DM* dermatomyositis

MHC associations revealed by genome-wide association studies (GWAS) can often not be fine-mapped to a single allele at a single locus; rather they comprise independent effects from multiple loci (see “Role of MHC variants in human diseases”). The presence of these multiple, independent effects highlights the heterogeneous nature within and between diseases, which may lead to varying immunological responses. Fine-mapping has also shown that autoimmune diseases share MHC alleles and hence molecular pathways, which are likely to represent targets for shared therapies. For instance, the major associations within MHC class II across autoimmune diseases imply that modulating T-cell receptor (TCR) activation by using peptide-bearing MHC molecules on antigen-presenting cells (APCs) could be therapeutically useful [17]. Shared MHC genetic factors have also been observed between autoimmune and infectious diseases, suggesting that human genetic architecture has evolved in response to natural selection as determined by various infectious pathogens [18].

### Advances in computational approaches for mapping MHC variation

Long-range LD between loci and SNP markers across the MHC offers an alternative approach to interrogate functional MHC variation through imputation. The development of different imputation tools using population-specific reference panels has enhanced the interpretation of genotype data derived from genome-wide platforms. MHC imputation is done using reference panels containing both genetic information and classic HLA serotyping, thus allowing identification of MHC allelic and amino acid variants. It is advantageous to impute allele and amino acid variants in the MHC because background sequence diversity causes the binary SNP concept to fail, technically speaking, while many SNPs have more than two alleles and various amino acids can be contained in the same position. For instance, six possible amino acid variants at position 11 in the HLA-DRB1 gene show the strongest association to rheumatoid arthritis (RA) [19]. Two of these (valine and leucine) confer susceptibility to RA, whereas the other four (asparagine, proline, glycine and serine) are protective.

Several tools allowing imputation of classic HLA alleles at four-digit resolution are now available for MHC imputation analysis; the most common are SNP2HLA [20], HLA*IMP:01 [21], and an improved HLA*IMP:02 [22]. HLA*IMP:02 outperforms HLA*IMP:01 on heterogeneous European populations and it increases the power and accuracy in cross-European GWAS [22]. Missing data are also better tolerated in HLA*IMP:02, while SNP genotyping platforms must be selected in HLA*IMP:01 [21, 22]. SNP2HLA not only imputes classic alleles but also amino acids by using two European reference panels, one based on data from HapMap-CEPH (90 individuals), and the other on the Type 1 Diabetes Genetics Consortium (T1DGC) study [20]. Another tool, HLA-VBSeq, allows imputation of MHC alleles at full resolution from whole-genome sequence data [23]. HLA-VBSeq does not require prior knowledge of MHC allele frequencies and can therefore be used for samples from genetically diverse populations [23]. It has successfully typed HLA-A alleles at full resolution in a Japanese population and identified rare causal variants implicated in complex human diseases [12].

One commonly used European reference panel for imputation is the T1DGC panel, which covers SNP genotyping and classic HLA serotyping information for 5225 unrelated individuals [20]. Similar population-specific reference panels have been developed for non-European studies to investigate the risk of psoriasis in Chinese populations [16] and of Graves’ disease and RA in Japanese populations. The panels have also been used to impute MHC alleles and amino acids for East Asian and Korean populations [24–26].

Using a single reference genome for regions like the MHC, which has substantial sequence and structural diversity, results in poor characterization. To counteract this, an algorithm was developed to infer much of the variation in the MHC; it allows genome inference from high-throughput sequencing data using known variation represented in a population reference graph (PRG) [27]. Specifically, the PRG constructed for the MHC combined eight assembled haplotypes, the sequences of known classic HLA alleles, and 87,640 SNP variants from the 1000 Genomes Project [28]. This approach is considered to be an intermediate step between de novo assembly and mapping to a single reference, but requires careful attention to the variation included in the PRG [27].

Despite the development of new tools to investigate MHC variation, the robustness of imputation depends largely on the reference panel and SNP selection. The frequency of alleles can differ between populations, thus highlighting the need to use population-specific reference panels to impute MHC alleles and amino acids. Additionally, the use of many samples is possible for analyzing the non-additive effects of MHC alleles on a wide scale, as described by Lenz et al. for celiac disease (CeD), psoriasis, and type 1 diabetes (T1D) [29]. These non-additive effects could explain our inability to identify susceptibility variants. However, one important limitation of existing imputation methods is that they are limited to the classic MHC alleles and their amino acids. Another limitation is that accuracy is lower for low frequency or rare variants [20, 30]; this can be improved by increasing the reference panel size, together with the use of deep sequencing data. Ascertainment bias and lower LD also make it challenging to impute MHC variants in some non-European populations, such as Africans.

MHC genetic variation mediates susceptibility to a wide range of complex diseases, including infectious and autoimmune diseases. The large volume of data generated by recent GWAS has provided an excellent opportunity to apply imputation tools used to fine-map MHC associations to classic alleles and amino acids, as described below for autoimmune diseases. Overall, MHC imputation has proved to be a robust and cost-effective way to identify causal genes underlying disease pathogenesis. Ultimately, knowing the causal genes will help explain disease heritability and lead to a better understanding of the molecular pathways involved in disease pathogenesis. Such work helps to pinpoint potential therapeutic targets.

## Role of MHC variants in human diseases

### Insights into MHC susceptibility for autoimmune diseases: fine-mapping results, epistasis, and disease biology

Associations between the MHC and autoimmune diseases reported in the 1970s were some of the earliest described genetic associations [31, 32], and they remain the strongest risk factors for autoimmune diseases. After the development of wide-screen genotyping platforms and imputation pipelines, MHC imputation and fine-mapping were performed in European and Asian populations for most common autoimmune diseases, including RA [19, 25, 33, 34], CeD [35], psoriasis [36], ankylosing spondylitis (AS) [37], systemic lupus erythematosus (SLE) [33, 38–41], T1D [42, 43], multiple sclerosis (MS) [44, 45], Graves’ disease [24], inflammatory bowel disease (IBD) [46], and dermatomyositis (DM) [47]. Table 1 shows the main associated variants and independently associated loci for autoimmune diseases.

In 2012, a pioneering MHC fine-mapping study, performed in individuals of European ancestry with RA [19], confirmed the strongest association with the class II HLA-DRB1 gene, as well as other independent associations. Previously an increased risk of RA was reported for a set of consensus amino acid sequences at positions 70–74 in the HLA-DRB1 gene, known as the “shared epitope” locus [48]. The imputed data revealed the most significant associations were with two amino acids at position 11, located in a peptide-binding groove of the HLA-DR heterodimer. This suggested a functional role for this amino acid in binding the RA-triggering antigen. Similar fine-mapping studies followed for other autoimmune diseases (Table 1).

In general, in most autoimmune diseases, fine-mapping strategies have confirmed the main associated locus reported by serotype analysis within a certain MHC locus. Such strategies have also allowed identification of specific allelic variants or amino acids, as well as independent variants in different HLA classes. For instance, in CeD, the strongest association was with the known DQ-DR locus, and five other independent signals in classes I and II were also identified. CeD is the only autoimmune disease for which the antigen, gluten, is known and well studied. Gluten is a dietary product in wheat, barley, and rye. It is digested in the intestine and deamidated by tissue transglutaminase enzymes such that it perfectly fits the binding pockets of a particular CeD-risk DQ heterodimer (encoded by the DQ2.2, DQ2.5, and DQ8 haplotypes). This association was confirmed by MHC fine-mapping, which indicated roles for four amino acids in the DQ genes with the strongest independent associations to CeD risk [35]. Similarly, the main associations were determined for T1D, MS, and SLE within the MHC class II locus (the associations for these three diseases are to a particular HLA-DQ-DR haplotype), and there are also independent, but weaker associations with the class I and/or III regions. In DM, fine mapping in an Asian population identified MHC associations driven by variants located around the MHC class II region, with HLA-DP1*17 being the most significant [47]. In contrast, the primary and strongest associations in psoriasis and AS were to MHC class I molecules, while independent associations to the class I locus were also reported for IBD and Graves’ disease. Class III variants are weakly implicated in autoimmune diseases, but several associations in the MHC class III region were seen for MS; for instance, the association to rs2516489 belonging to the long haplotype between *MICB* and *LST1* genes. The association signal to rs419788-T in the class III region gene *SKIV2L* has also been implicated in SLE susceptibility, representing a novel locus identified by fine-mapping in UK parent–child trios [39]. An independent association signal to class III was also identified (rs8192591) by a large meta-analysis of European SLE cases and controls and, specifically, upstream of *NOTCH4* [40]. However, further studies are needed to explain how these genetic variations contribute to predisposition to SLE.

In addition to identifying independent variants, MHC fine-mapping studies permit analysis of epistatic and non-additive effects in the locus. These phenomena occur when the effect of one allele on disease manifestation depends on the genotype of another allele in the locus (non-additive effect), or on the genotype of the “modifier” gene in another locus (epistasis). Non-additive MHC effects were established in CeD, in which knowing gluten was the causal antigen offered an advantage in investigating the antigen-specific structure of the DQ-heterodimer. CeD risk is mediated by the presence of several HLA-DQ haplotypes, including the DQ2.5, DQ2.2, and DQ8 haplotypes, which form the specific pocket that efficiently presents gluten to T cells. These haplotypes can be encoded either in *cis*, when both DQA1 and DQB1 are located on the same chromosome, or *in trans*, when they are located on different chromosomes. Some DQ allelic variants confer susceptibility to CeD only in combination with certain other haplotypes, forming a CeD-predisposing *trans*-combination. For example, HLA-DQA1*0505-DQB1*0301 (DQ7) confers risk to CeD only if it is combined with DQ2.2 or DQ2.5, contributing to the formation of susceptible haplotypes *in trans*. In particular, DQ7/DQ2.2 heterozygosity confers a higher risk for CeD than homozygosity for either of these alleles, and is an example of a non-additive effect for both alleles.

Unlike CeD, the exact haplotypes and their associated properties remain unknown for most other autoimmune diseases; therefore, analyzing non-additive effects might yield new insights into potentially disease-causing antigens. Lenz et al. provided evidence of significant non-additive effects for autoimmune diseases, including CeD, RA, T1D, and psoriasis, which were explained by interactions between certain classic HLA alleles [29]. For instance, specific interactions that increase T1D disease risk were described between HLA-DRB1*03:01-DQB1*02:01/DRB1*04:01-DQB1*03:02 genotypes [49] and for several combinations of the common HLA-DRB1, HLA-DQA1, and HLA-DQB1 haplotypes [43]. In AS, epistatic interaction was observed for combinations of HLA-B60 and HLA-B27, indicating that individuals with the HLA-B27+/HLA-B60+ genotype have a high risk of developing AS [50]. Moreover, a recent study in MS found evidence for two interactions involving class II alleles: HLA-DQA1*01:01-HLA-DRB1*15:01 and HLA-DQB1*03:01-HLA-DQB1*03:02, although their contribution to the missing heritability in MS was minor [44].

Epistatic interactions between MHC and non-MHC alleles have also been reported in several autoimmune diseases, including SLE, MS, AS, and psoriasis. For instance, in a large European cohort of SLE patients, the most significant epistatic interaction was identified between the MHC region and cytotoxic T lymphocyte antigen 4 (*CTLA4*) [9], which is upregulated in T cells upon encountering APCs. This highlights that appropriate antigen presentation and T-cell activation are important in SLE pathogenesis [9]. Notably, interactions between MHC class I and specific killer immunoglobulin receptor (KIR) genes are important in predisposition to autoimmune diseases such as psoriatic arthritis, scleroderma, sarcoidosis, and T1D [51–54]. KIR genes are encoded by the leukocyte receptor complex on chromosome 19q13 and expressed on natural killer cells and subpopulations of T cells [55]. Finally, epistatic interactions between MHC class I and *ERAP1* have been described for AS, psoriasis, and Behçet’s disease [10].

Association of novel MHC variants and identification of interaction effects within the MHC are increasing our understanding of the biology underlying autoimmune and inflammatory diseases. Fine-mapping the main associated locus within HLA-DQ-DR haplotypes has allowed determination of the key amino acid positions in the DQ or DR heterodimer. Pinpointing specific amino acids leads to a better understanding of the structure and nature of potential antigens for autoimmune or inflammatory diseases, and these can then be tested through binding assays and molecular modeling. The fact that these positions are located in peptide-binding grooves suggests they have a functional impact on antigenic peptide presentation to T cells, either during early thymic development or during peripheral immune responses [19]. In addition, analysis of non-additive effects in MHC-associated loci offers the possibility to identify antigen-specific binding pockets and key amino acid sequences. For example, identification of the protective, five-amino acid sequence DERAA as a key sequence in the RA-protective HLA-DRB1:13 allele, and its similarity to human and microbial peptides, led to identification of (citrullinated) vinculin and some pathogen sequences as novel RA antigens [56].

The identification of independent signals in MHC classes I and III for many autoimmune diseases implies that these diseases involve novel pathway mechanisms. For example, association of CeD to class I molecules suggests a role for innate-like intraepithelial leucocytes that are restricted to class I expression and that are important in epithelial integrity and pathogen recognition [57]. Class I associations to RA, T1D, and other autoimmune diseases suggest that CD8^+^ cytotoxic cells are involved in disease pathogenesis, as well as CD4^+^ helper T cells.

Discovering the epistatic effects of MHC and non-MHC loci can also shed light on disease mechanisms. For example, *ERAP1* loss-of-function variants reduce the risk of AS in individuals who are HLA-B27-positive and HLAB-40:01-positive, but not in carriers of other risk haplotypes [37]. Similar epistatic effects were also observed for psoriasis, such that individuals who carry variants in ERAP1 showed an increased risk only when they also carried an HLA-C risk allele [58]. In line with these observations, mouse studies have shown that *ERAP1* determines the cleavage of related epitopes in such a way that they can be presented by the HLA-B27 molecule [37]. Confirming that certain epitopes must be cleaved by ERAP1 to be efficiently presented by CD4^+^ and CD8^+^ cells will be a critical step in identifying specific triggers for autoimmune diseases.

The recent discoveries of genetic associations between MHC alleles and autoimmune diseases are remarkable and offer the potential to identify disease-causing antigens. This would be a major step towards developing new treatments and preventing disease. However, we still do not understand exactly how most associated alleles and haplotypes work, and extensive functional studies are needed to clarify their involvement in disease.

### Explained heritability by independent MHC loci for autoimmune diseases

Heritability is an estimation of how much variation in a disease or phenotype can be explained by genetic variants. Estimating heritability is important for predicting diseases but, for common diseases, it is challenging and depends on methodological preferences, disease prevalence, and gene–environment interactions that differ for each phenotype [59]. It is therefore difficult to compare heritability estimates across diseases. Nevertheless, for many diseases, estimates have been made as to how much phenotypic variance can be explained by the main locus and by independent MHC loci [29].

For autoimmune diseases with a main association signal coming from a class II locus, the reported variance explained by MHC alleles varies from 2 − 30% [9]. The strongest effect is reported for T1D, in which the HLA-DR and HLA-DQ haplotypes explain 29.6% of phenotypic variance; independently associated loci in HLA-A, HLA-B, and HLA-DPB1 together explain about 4% of the total phenotypic variance, while all other non-MHC loci are responsible for 9% [60]. Similarly, in CeD, which has the same main associated haplotype as T1D, the HLA-DQ-DR locus explains 23 − 29% of disease variance (depending on the estimated prevalence of disease, which is 1 − 3%), whereas other MHC alleles explain 2 − 3%, and non-MHC loci explain 6.5 − 9% [35]. In seropositive RA, 9.7% of phenotypic variance is explained by all the associated DR haplotypes, whereas a model including three amino acid positions in DRB1, together with independently associated amino acids in HLA-B and HLA-DP loci, explains 12.7% of the phenotypic variance [19]. This indicates that non-DR variants explain a proportion of heritability comparable to that in other non-MHC loci (4.7 − 5.5% in Asians and Europeans) [19]. The non-additive effects of DQ-DR haplotypes can also explain a substantial proportion of phenotypic variance: 1.4% (RA), 4.0% (T1D), and 4.1% (CeD) [29]. In MS, the major associated allele, DRB1*15:01, accounts for 10% of the phenotypic variance, whereas all the alleles in DRB1 explain 11.6%. A model including all of the independent variants (and those located in classes I, II, and III) explains 14.2% of the total variance in MS susceptibility [45].

In SLE, the proportion of variance explained by the MHC is notably lower, at only 2% [41], and is mostly due to class II variants. In IBD, the association with MHC is weaker than in classic autoimmune diseases, with a lower contribution seen in Crohn’s disease (CD) than in ulcerative colitis (UC) [61]. The main and secondary variants can now explain 3.1% of heritability in CD and 6.2% in UC, which is two to ten times greater than previously attributed by main effect analysis in either disease (0.3% in CD and 2.3% in UC for the main SNP effect) [46]. Among all the diseases discussed here, the main effect of the associated haplotype is far stronger than the independent effects from other loci (with the exception of IBD, in which the MHC association is weaker overall). However, independent MHC loci can now explain a comparable amount of the disease variance to that explained by the non-MHC associated genes known so far.

### Insights into MHC susceptibility for infectious diseases: GWAS, fine-mapping results, and epistasis

In principle, an infectious disease is caused by interactions between a pathogen, the environment, and host genetics. Here, we discuss MHC genetic associations reported in infectious diseases from GWAS (Table 2) and how these findings can explain increased susceptibility or protection by affecting human immune responses. This is why certain MHC classes are important in infectious diseases. We note that fewer MHC associations have been found for infectious diseases than for autoimmune diseases, mainly because of the smaller cohort sizes for infectious diseases. Thus, extensive fine-mapping studies (and imputation) have yet to be performed, with the exception of a few studies on infections such as human immunodeficiency virus (HIV) [62], human hepatitis B virus (HBV) [63, 64], human hepatitis C virus (HCV) [65], human papilloma virus (HPV) seropositivity [66], and tuberculosis [67].

**Table 2:** Major histocompatibility complex (MHC) associations and risks for infectious diseases identified by genome-wide association studies (GWAS)

Phenotype	Initial sample size	Replication sample size	Mapped / nearest gene	SNPs	*P*-value	OR or beta	Reference
HIV-1 viral set point	Euro-CHAVI, 486 seroconverters	140 Caucasian patients	HCP5/B*5701	rs2395029	9.36 × 10^−12^	NA	[69]
			HLA-C	rs9264942	3.77 × 10^−9^	NA	
HIV-1 control	2362 European ancestry cases	NA	HSPA1B-C6orf48 LOC105375015 LOC105375015 HCP5 HCP5 PSORS1C1	rs9368699 rs9264942 rs9264942 rs2395029 rs2395029 rs3815087	5.00 × 10^–08^ 6.00 × 10^–32^ 6.00 × 10^–12^ 5.00 × 10^–35^ 1.00 × 10^–11^ 8.00 × 10^–08^	NA NA NA NA NA NA	[70]
AIDS progression	275 seropositive non-progressors, 86 seropositive rapid progressors, 1352 seronegative controls, All Europeans	See [69]	HCP5	rs2395029	3.00 × 10^–19^		[73]
			LOC105375015	rs10484554	6.00 × 10^–08^	NA	
HIV-1 control	974 controllers (cases) and 2648 progressors (controls) multi ethnic (Europeans, African-Americans, and Hispanics)	Untreated HIV-infected persons from Switzerland	HLA-C	rs9264942	2.8 × 10^−35^	2.9	[62]
			HLA-B*57:01	rs2395029	9.7 × 10^−26^	5.3	
			MICA	rs4418214	1.4 × 10^−34^	4.4	
			PSORS1C3	rs3131018	4.2 × 10^−16^	2.1	
			HLA-B	rs2523608	8.9 × 10^–20^	2.6	
			Intergenic	rs2255221	3.5 × 10^–14^	2.7	
			HLA-B	rs2523590	1.7 × 10^–13^	2.4	
HIV-1 susceptibility	6334 cases, 7247 controls (Europeans)	NA	MICA - LOC105375017	rs4418214	4.00 × 10^–11^	1.52	[72]
Chronic hepatitis B infection	786 Japanese cases, 2201 Japanese controls	Japanese and Thai cohorts of 1300 cases and 2100 controls	HLA-DPA1	rs3077	2.31 × 10^–38^	0.56	[80]
			HLA-DPB1	rs9277535	6.34 × 10^–39^	0.57	
Chronic hepatitis B infection	458 Japanese cases, 2056 Japanese controls	Three independent Japanese cohorts (2209 cases and 4440 controls)	HLA-DPA1	rs3077	1.28 × 10^−61^	1.98	[83]
			HLA-DPB1	rs9277535	3.72 × 10^−17^	1.95	
			HLA-DQB1	rs2856718	4.41 × 10^−10^	1.59	
			HLA-DQB2	rs7453920	1.27 × 10^−10^	2.2	
Chronic hepatitis B infection	400 cases, 1000 controls, Koreans	971 cases, 1938 controls Korean	TCF19	rs1419881	1.00 × 10^–18^	1.37	[77]
			EHMT2	rs652888	7.00 × 10^–13^	1.38	
			HLA-DQB1 - LOC102725019	rs2856718	2.00 × 10^–24^	1.6	
			HLA-DQB2	rs7453920	7.00 × 10^–26^	2	
			HLA-DPA1	rs3077	5.00 × 10^–39^	1.89	
			HLA-DPB1	rs9277535	4.00 × 10^–40^	1.89	
Hepatitis B	951 carrier cases, 937 cleared controls, Han Chinese	4230 carrier cases, 3051 cleared controls, 2622 controls, Han Chinese	HCG27 - HLA-C	rs3130542	9.00 × 10^–14^	1.33	[76]
			HLA-DQB2	rs7453920	5.00 × 10^–37^	1.8868	
Chronic hepatitis B infection	321 cases, 304 controls, TaiwaneseHan Chinese	1302 cases, 761 controls, Taiwanese Han Chinese	LOC102725019 - HLA-DQA2	rs9276370	2.00 × 10^–12^	1.95	
			HLA-DQB2	rs7453920	7.00 × 10^–15^	2.31	[79]
			HLA-DPB1	rs9277535	5.00 × 10^–14^	1.59	
			LOC105375021	rs9366816	3.00 × 10^–10^	1.43	
			HLA-DQB1 - HLA-DQA2; LOC102725019 - HLA-DQA2; LOC102725019 - HLA-DQA2; LOC102725019 - HLA-DQA2; HLA-DQB1 - HLA-DQA2; LOC102725019 - HLA-DQA2; LOC102725019 - HLA-DQA2; LOC102725019 - HLA-DQA2, HLA-DQB2; HLA-DQB2; HLA-DQB2; HLA-DQB2; HLA-DQB2; HLA-DQB2; HLA-DQB2; HLA-DQB2, HLA-DPB1; HLA-DPB1; HLA-DPB1; HLA-DPB1; HLA-DPB1; HLA-DPB1; HLA-DPB1; HLA-DPB1, LOC105375021; LOC105375021	rs9276370, rs7756516, rs7453920, rs9277535, rs9366816	1.00 × 10^–12^	1.49	
Chronic hepatitis B infection	2514 cases, 1130 controls, Chinese	6600 cases, 8127 controls, Chinese	HCG27 - HLA-C	rs2853953	5.00 × 10^–20^	1.47	[78]
			CFB	rs12614	1.00 × 10^–34^	1.89	
			NOTCH4	rs422951	5.00 × 10^–16^	1.27	
			HLA-DQB1 - LOC102725019	rs2856718	7.00 × 10^–28^	1.28	
			HLA-DQB2	rs7453920	1.00 × 10^–60^	2	
			HLA-DOA	rs378352	1.00 × 10^–23^	1.26	
			HLA-DPA1	rs3077	1.00 × 10^–53^	1.45	
			HLA-DPB1	rs9277535	1.00 × 10^–70^	1.52	
Chronic hepatitis B infection/progression	181 Japanese carriers, 184 Japanese healthy controls	256 carriers, 236 healthy controls (Japanese) and 344 carriers, 151 healthy controls (Korean)	3' UTR of HLA-DPB1	rs9277542	8.36 × 10^–8^	0.42	[81]
Chronic hepatitis C infection	1482 chronic cases, 919 spontaneously cleared cases, European, African, and mixed/other ancestry	461 chronic cases, 284 spontaneously cleared cases	HLA-DQB1 - LOC102725019	rs4273729	5.00 × 10^–17^	1.59	[65]
Chronic hepatitis C infection	481 cases, 2963 controls, Japanese	5737 cases, 26,931 controls Japanese	HLA-DQB1 - LOC102725019	rs9275572	4.00 × 10^–16^	1.27	[84]
HPV seropositivity	1286 lung cancer cases, 679 head and neck cancer cases, 811 kidney cancer cases, 2035 controls, European ancestry	1307 head and neck cancer cases, 1037 controls, Hispanic	HLA-DQB1 - LOC102725019	rs9357152	1.00 × 10^–14^	2.02	[85]
Dengue shock syndrome	2008 child cases, 2018 child controls, Vietnamese	1737 child cases, 2,934 child controls, Vietnamese	MICB	rs3132468	4.00 × 10^–11^	1.34	[74]
Leprosy	706 cases, 1225 controls Han Chinese	3254 cases, 5955 controls, Chinese	HLA-DRB1 - HLA-DQA1	rs602875	5.00 × 10^–27^	1.61	[88]
Leprosy	1548 cases, 2150 controls, 4362 controls with immune-related diseases, Chinese ancestry	6765 cases, 9505 controls, Chinese ancestry	HLA-DRB1 - HLA-DQA1	rs9271100	8.00 × 10^–95^	1.68	[87]
*M. Tuberculosis* infection	8162 cases, 277,643 controls, Icelanders	5530 Russian cases, 5607 Russian controls and 438 cases from Croatia, 1009 controls from Croatia	HLA-DQA1, HLA-DRB1	rs557011	3.1 × 10^−13^	1.14	[67]
Pulmonary tuberculosis			HLA-DQA1, HLA-DRB1	rs557011	5.8 × 10^−12^	1.25	
Pulmonary tuberculosis			HLA-DQA1, HLA-DRB1	rs9271378	2.5 × 10^−12^	0.78	
*M. Tuberculosis* infection			HLA-DQA1	rs9272785	9.3 × 10^−9^	1.14	
Leishmaniasis (visceral)	989 cases, 1089 controls (Indians), 357 cases, 1613 unaffected relatives (Brazilians)	951 cases, 990 controls, Indians	HLA-DRB1 - HLA-DQA1	rs9271858	3.00E^–17^	1.41	[92]

Only genome-wide significant single nucleotide polymorphisms (SNPs) are given (*P* > 5 × 10^–8^), which are located in the MHC region (chr6:28,477,797-33,448,354). Assembly GRCh37/hg19

*AIDS* acquired immune deficiency syndrome, *NA* not applicable, *OR* odds ratio

From a genetic viewpoint, one of the best-studied infectious diseases is HIV infection. MHC class I loci have strong effects on HIV control [62, 68–71] and acquisition [72], viral load set point [69–71], and non-progression of disease [73] in Europeans [69, 70, 72, 73], and in multi-ethnic populations (Europeans, African-Americans, Hispanics, and Chinese) [62, 68, 71]. A GWAS of an African-American population indicated a similar HIV-1 mechanism in Europeans and African-Americans: about 9.6% of the observed variation in viral load set point can be explained by HLA-B*5701 in Europeans [69], while about 10% can be explained by HLA-B*5703 in African-Americans [68]. In contrast, the MHC associations and imputed amino acids identified in Europeans and African-Americans were not replicated in Chinese populations, possibly because of the varied or low minor allele frequencies of these SNPs in Chinese people [71]. A strong association to the MHC class I polypeptide-related sequence B (*MICB*) was also revealed by a recent GWAS for dengue shock syndrome (DSS) in Vietnamese children [74]. This result was replicated in Thai patients, indicating *MICB* can be a strong risk factor for DSS in Southeastern Asians [75].

HLA-DP and HLA-DQ loci, along with other MHC or non-MHC loci (TCF19, EHMT2, HLA-C, HLA-DOA, UBE2L3, CFB, CD40, and NOTCH4) are consistently associated with susceptibility to HBV infection in Asian populations [76–83]. Significant associations between the HLA-DPA1 locus and HBV clearance were also confirmed in independent East Asian populations [79, 81]. A fine-mapping study of existing GWAS data from Han Chinese patients with chronic HBV infection used SNP2HLA as the imputation tool and a pan-Asian reference panel. It revealed four independent associations at HLA-DPβ1 positions 84–87, HLA-C amino acid position 15, rs400488 at HCG9, and HLA-DRB1*13; together, these four associations could explain over 72.94% of the phenotypic variance caused by genetic variations [64]. Another recent study using imputed data from Japanese individuals indicated that class II alleles were more strongly associated with chronic HBV infection than class I alleles (Additional file 1) [63]. Similarly, the HLA-DQ locus influences the spontaneous clearance of HCV infection in cohorts of European and African ancestry, while DQB1*03:01, which was identified by HLA genotyping together with the non-MHC IL28B, can explain 15% of spontaneous HCV infection clearance cases [65]. HLA-DQB1*03 also confers susceptibility to chronic HCV in Japanese people [84]. A GWAS in a European population revealed that HPV8 seropositivity is influenced by the MHC class II region [85]. However, HPV type 8 showed a higher seropositivity prevalence than other HPV types at the population level [66]; this led to a limited power to detect associations with other HPV types. Fine-mapping using the same European population as in the GWAS [66] revealed significant associations with HPV8 and HPV77 seropositivity, but only with MHC class II alleles, not with class I alleles. This indicates a pivotal role for class II molecules in antibody immune responses in HPV infection. Notably in this study, imputation was performed using HLA*IMP:02 and reference panels from the HapMap Project [86] and the 1958 British Birth Cohort, as well as using SNP2HLA with another reference panel from the T1DGC. Both imputation tools provided comparable results, thus highlighting the important role of MHC class II alleles in antibody response to HPV infection [66].

A GWAS on leprosy in Chinese populations pointed to significant associations with HLA-DR-DQ loci [87, 88]; these results were replicated in an Indian population [89]. Fine-mapping the MHC showed that variants in HLA class II were extensively associated with susceptibility to leprosy in Chinese people, with HLA-DRB1*15 being the most significant variant [87]. HLA class II variants also influence the mycobacterial infection tuberculosis in European and African populations [67, 90]. Fine-mapping identified the DQA1*03 haplotype, which contains four missense variants and contributes to disease susceptibility [67]. A meta-analysis showed that five variants (HLA-DRB1*04, *09, *10, *15, and *16) increase the risk of tuberculosis, especially in East Asian populations, whereas HLA-DRB1*11 is protective [91].

Using a population from Brazil, the first GWAS on visceral leishmaniasis revealed that the class II HLA-DRB1-HLA-DQA1 locus had the strongest association signal; this was replicated in an independent Indian population [92]. This common association suggests that Brazilians and Indians share determining genetic factors that are independent of the different parasite species in these geographically distinct regions.

Finally, epistatic interactions between MHC class I alleles and certain KIR alleles (between KIR3DS1 combined with HLA-B alleles) are associated with slower progression to acquired immunodeficiency syndrome (AIDS) [93] and better resolution of HCV infection (between KIR2DL3 and its human leukocyte antigen C group 1, HLA-C1) [94].

### Insights into the biology of infectious diseases

Associations with the MHC class I locus suggest a critical role for CD8^+^ T-cell responses in major viral infections such as HIV, dengue, and HCV. This critical role of CD8^+^ T-cell responses in HIV infection is reflected by the slow disease progression seen in infected individuals because of their increasing CD8^+^ T-cell responses that are specific to conserved HIV proteins such as Gap p24 [95]. Interestingly, five out of six amino acid residues (Additional file 1) identified as associated with HIV control [62] lie in the MHC class I peptide-binding groove, implying that MHC variation affects peptide presentation to CD8^+^ T cells. In particular, the amino acid at position 97, which lies in the floor of the groove in HLA-B, was most significantly associated with HIV control (*P* = 4 × 10^−45^) [62]. This amino acid is also implicated in MHC protein folding and cell surface expression [96]. An association found in severe dengue disease also underscores the role of CD8^+^ T cells in disease pathogenesis: class I alleles that were associated with an increased risk of severe dengue disease were also associated with weaker CD8^+^ T-cell responses in a Sri Lankan population from an area of hyper-endemic dengue disease [97]. In HCV, similar to the protective alleles against HIV infection [95], HLA-B*27 presents the most conserved epitopes of HCV to elicit strong cytotoxic T-cell responses, thereby reducing the ability of HCV to escape from host immune responses [98].

Associations between genetic variants in the MHC class II region and disease susceptibility imply that impaired antigen presentation or unstable MHC class II molecules contribute to insufficient CD4^+^ T-cell responses and, subsequently, to increased susceptibility to infections. For instance, the amino acid changes at positions of HLA-DPβ1 and HLA-DRβ1 in the antigen-binding groove that influence HBV infection may result in defective antigen presentation to CD4^+^ T cells or to impaired stability of MHC class II molecules, thereby increasing susceptibility to HBV infection [64]. CD4^+^ T-cell responses are also critical in mycobacterial infections, such as has been described for leprosy and tuberculosis [99, 100]. Notably, monocyte-derived macrophages treated with live *Mycobacterium leprae* showed three main responses that explain infection persistence: downregulation of certain pro-inflammatory cytokines and MHC class II molecules (HLA-DR and HLA-DQ), preferentially primed regulatory T-cell responses, and reduced Th1-type and cytotoxic T-cell function [99]. Macrophages isolated from the lesions of patients with the most severe disease form, lepromatous leprosy, also showed lower expression of MHC class II molecules, providing further evidence that defective antigen presentation by these molecules leads to more persistent and more severe *M. leprae* infection [99].

Recently, it has been shown that CD4^+^ T-cells are essential for the optimal production of IFNγ by CD8^+^ T-cells in the lungs of mice infected with *M. tuberculosis*, indicating that communication between these two distinct effector cell populations is critical for a protective immune response against this infection [101]. Impaired antigen processing and presentation from *Leishmania*-infected macrophages (which are the primary resident cells for this parasite) to CD4^+^ T cells could explain increased susceptibility to leishmaniasis [102]. The association between HPV seropositivity and the MHC class II region also suggests that class II molecules bind and present exogenous antigens more effectively to a subset of CD4^+^ T cells known as Th2. These Th2 cells help primed B lymphocytes to differentiate into plasma cells and to secrete antibodies against the HPV virus.

In support of the hypothesis that genetic effects on both CD8^+^ (class I) and CD4^+^ (class II) cells modify the predisposition to infections, it should be noted that some infectious diseases, such as HIV, HBV, HCV, and leprosy, show associations to more than one of the classic MHC classes and, in some cases, the associations differ between populations (Table 2). Moreover, consideration must be given to the differences between viral and bacterial genotypes in the same infection, which play a role in determining potentially protective effects. Overall, associations with multiple MHC loci reflect the complex and interactive nature of host immune responses when the host encounters a pathogen.

## Relationship between the MHC variants involved in autoimmune and infectious diseases

**Fig. 2:**
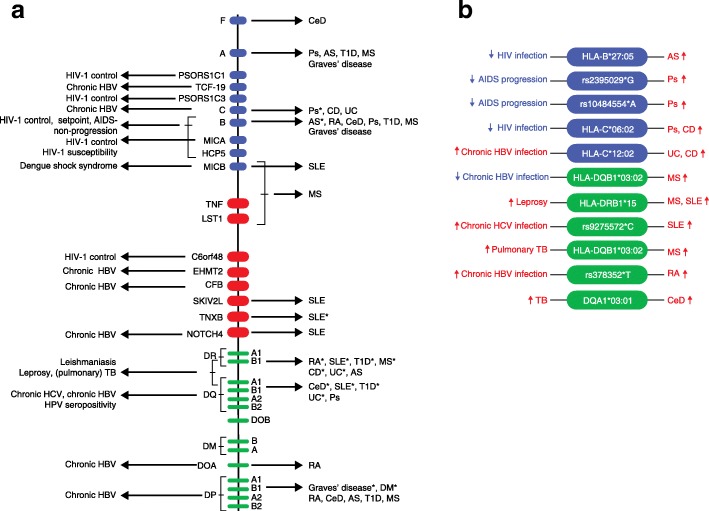
Major histocompatibility complex allele associations with autoimmune and infectious diseases. **a** Abbreviations marked with an asterisk indicate the autoimmune disease showing the strongest association with the specific locus. **b** Single nucleotide polymorphisms (SNPs) and alleles in the major histocompatibility complex (MHC) shared between autoimmune and infectious diseases. The *blue area* shows MHC alleles located in the class I region and the *green area* shows those in the class II region. The *blue arrows* indicate either a protective effect of the genetic variants against the infectious disease or a slower progression to the infectious disease. The *red arrows* indicate increased susceptibility to the corresponding autoimmune or infectious disease. *AIDS* acquired immunodeficiency syndrome, *AS* ankylosing spondylitis, *CD* Crohn’s disease, *CeD* celiac disease, *DM* dermatomyositis, *HBV* hepatitis B virus, *HCV* hepatitis C virus, *HIV* human immunodeficiency virus, *MS* multiple sclerosis, *Ps* psoriasis, *RA* rheumatoid arthritis, *SLE* systemic lupus erythematosus, *T1D* type 1 diabetes, *TB* tuberculosis, *UC* ulcerative colitis, *HPV* human papilloma virus

Both autoimmune and infectious diseases seem to involve certain MHC classes (Fig. 2a), and only a few MHC alleles are shared between these two distinct disease groups (Fig. 2b). The identification of shared MHC variation has provided insight into the relationships between the MHC variants involved in autoimmune and infectious diseases and which have been uniquely shaped throughout human evolution [18].

Two hypotheses have been proposed to explain the relationships between the MHC variants involved in both groups of diseases. The first, known as the “pathogen-driven selection” hypothesis, states that pressure exerted on the human genome by pathogens has led to the advantageous selection of host defense genes and, subsequently, to much higher polymorphism in the MHC. This polymorphism has contributed to the development of complex immune defense mechanisms that protect humans against a broad range of pathogens. Thus, heterozygosity at MHC loci is evolutionarily favored and has become an efficient mechanism contributing to the highly polymorphic MHC (the “MHC heterozygosity advantage”) [103]. Two examples of MHC heterozygote advantage are HIV-1-infected heterozygotes at class I loci, which are slower to progress to AIDS [104, 105], and HBV-infected heterozygotes at class II loci, which seem more likely to clear the infection [106]. In addition, human populations exposed to a more diverse range of pathogens display higher class I genetic diversity than those exposed to a smaller range [107]. However, the true effect of infectious diseases on selection might be underestimated because of the heterogeneity of many pathogens and the changing prevalence of infectious diseases over evolutionary time.

Positive selection of the advantageous effect of MHC polymorphism in infections may also be accompanied by a higher risk of developing autoimmune diseases. For example, the non-MHC locus *SH2B3* rs3184504*A is a risk allele for CeD but has been under positive selection because it offers the human host protection against bacterial infections [108]. To investigate whether other genetic variants in the MHC show this opposite direction effect between autoimmune and infectious diseases (Fig. 2b), we compared SNPs and alleles in the MHC identified by GWAS and fine-mapping studies on autoimmune diseases (Table 1; Additional file 2) with those identified in infectious diseases (Table 2; Additional file 1). On the one hand, HLA-B*27:05, which has one of the strongest associations to AS in the MHC (*P* < 1 × 10^−2000^) [37] and is present in all ethnic groups, increases AS risk. On the other hand, it also has a protective effect against HIV infection, showing a nominal significant value of 5.2 × 10^–5^ [70]. The second example of opposite allelic effect is the association between the rs2395029*G allele and susceptibility to psoriasis (OR = 4.1; *P* = 2.13 × 10^–26^) [109] and AIDS non-progression (*P* = 9.36 × 10^–12^) [69]. Located in the HLA complex P5 (HCP5), rs2395029 is a proxy for the HLA-B*57:01 allele [69], the strongest protective allele against AIDS progression [110]. Non-progressors carrying the rs2395029-G allele had a lower viral load than other non-progressors [73].

Another study showed that psoriasis patients carry the same genetic variants as HIV controllers/non-progressors and that they are particularly enriched for the protective allele HLA-B*57:01 (*P* = 5.50 × 10^–42^) [111]. Moreover, the intergenic variant rs10484554*A, which is in LD with HLA-C (r^2^ ≥ 0.8), was significantly associated with AIDS non-progression (*P* = 6.27 × 10^–8^) [73] and with susceptibility to psoriasis (OR = 4.66, *P* = 4 × 10^–214^) [58]. HLA-C*06:02 (equivalent to HLA-Cw6) was most strongly associated with susceptibility to psoriasis (OR = 3.26; *P* = 2.1 × 10^–201^) [36] and is also protective against HIV infection (OR = 2.97; *P* = 2.1 × 10 ^–19^) [62]. The same allele has been associated with susceptibility to CD (OR = 1.17; *P* = 2 × 10^–13^) [46]. Interestingly, the role of MHC in HIV control also relates to the influence of MHC expression levels. For instance, rs9264942 shows one of the most significant genome-wide effects observed on HIV control [62, 69, 70]: it is located 35 kb upstream of the HLA-C locus (Table 2) and has been associated with high HLA-C expression, conferring protection against HIV infection [112]. Explaining this protective effect, HLA-C allelic expression was correlated with increasing likelihood of CD8^+^ T-cell cytotoxicity [112]. However, the −35 SNP is not a causal variant, but is in LD with a SNP at the 3′ end of HLA-C; this affects HLA-C expression by influencing binding of the microRNA Hsa-miR-148a [113]. Notably, high HLA-C expression has a deleterious effect by conferring risk for Crohn’s disease [113]. The potential mechanism by which HLA expression levels confer resistance to pathogens, and also lead to greater autoimmunity, could be through promiscuous peptide binding [114]. Lastly, HLA-DQB1*03:02 showed a dominant risk effect for MS (OR = 1.30; *P* = 1.8 × 10^–22^) [45], whereas it is a resistant allele against chronic HBV infection (OR = 0.59; *P* = 1.42 × 10^–5^) [63].

The second hypothesis states that pathogens can trigger autoimmunity, as suggested by epidemiological studies [115, 116]. For example, it has recently been shown that apoptosis of infected colonic epithelial cells in mice induces the proliferation of self-reactive CD4^+^ T cells that are specific to cellular and to pathogenic antigens [117]. Self-reactive CD4^+^ T cells differentiate into Th17 cells, which promote production of auto-antibodies and auto-inflammation, implying that infections can trigger autoimmunity [117]. Other mechanisms have been proposed, such as molecular mimicry, bystander activation, exposure of cryptic antigens, and superantigens [118]. Common genetic signatures between autoimmune and infectious diseases indirectly imply that pathogens can indeed trigger autoimmunity. In line with this second hypothesis, we have identified common risk factors between autoimmune and infectious diseases, such as the alleles: HLA-DRB1*15 for MS, SLE (Table 1), and leprosy (OR = 2.11; *P* = 3.5 × 10^–28^) [87]; rs9275572*C, located in HLA-DQ, for chronic HCV infection (OR = 0.71; *P* = 2.62 × 10^–6^) [84], and SLE (*P* = 1.94 × 10^–6^) [119]; HLA-DQB1*03:02 for MS (OR = 1.30; *P* = 1.8 × 10^–22^) [45] and pulmonary tuberculosis (OR = 0.59; *P* = 2.48 × 10^–5^) [67]; HLA-C*12:02 for UC (OR = 2.25; *P* = 4 × 10^–37^) [46], CD (OR = 1.44; *P* = 3x 10^–8^) [46], and chronic HBV infection (OR = 1.70; *P* = 7.79 × 10^–12^) [63]; and rs378352*T, located in HLA-DOA, for chronic HBV infection (OR = 1.32; P = 1.16 × 10^–7^) [78] and RA (OR = 1.24; *P* = 4.6 × 10^–6^) [25] (Fig. 2a).

Associations within the MHC region for several autoimmune diseases, such as RA, CeD, AS, T1D, Graves’ disease, and DM, and HBV infection are driven by variants and alleles around HLA-DPB1 (Table 1), implying that viruses like HBV could trigger autoimmunity. Although there is no convincing evidence, HBV and HCV are associated with extra-hepatic autoimmune perturbations [120, 121]. Lastly, the DQA1*03:01 allele, which contributes to tuberculosis susceptibility (OR = 1.31; *P* = 3.1 × 10^–8^) [67], is also a well-known risk factor for CeD as part of the DQ8 (DQA1*03-DQB1*03:02) and DQ2.3 (trans-DQA1*03:01 and DQB1*02:01) haplotypes [122]. DQA1*03 also increases susceptibility to T1D, RA, and juvenile myositis [123–125]. Overall, the direction of association is the same for most shared MHC class II loci, suggesting that bacteria and viruses can trigger immune responses. No viruses have been proven to cause an autoimmune disease thus far, but multiple virus infections could prime the immune system and eventually trigger an autoimmune response; this is a hypothesis that has been supported by animal studies on MS [126].

## Conclusions and future perspectives

We have discussed recent advances in understanding the genetic variation in the MHC in relation to autoimmune and infectious diseases. However, confidence in the associations between MHC and infectious diseases is limited, mainly because of the relatively small patient cohort sizes available. Further limitations to identifying and replicating associations with infectious diseases include: strain differences, heterogeneity in clinical phenotypes, use of inappropriate controls (such as individuals with asymptomatic infections), and population-specific differences in allele frequency and/or haplotype structure. Finally, with the exception of a few described above, no imputation has been performed in most infectious disease studies. In certain populations, such as Africans, lower LD makes it challenging to perform MHC imputation.

Although application of a traditional GWAS is challenging for infectious diseases, other approaches may increase the power of genetic studies. For instance, a combination of transcriptional analysis and systems biology allowed the identification of a novel role for type I IFN signaling pathway in the human host immune response against *Candida albicans* [127]. The use of control subjects for whom it is known whether they clear the infection, and who come from the same hospital as patients, could be appropriate for infectious diseases so that co-morbidities and clinical risk factors are as similar as possible between groups. Overall, initiating collaborative efforts to increase patient cohort numbers, designing better studies by using more appropriate controls and more homogenously clinically defined patient phenotypes, and applying imputation using population-specific reference genomes would open new avenues to study the genetics of infectious diseases.

In contrast to infectious diseases, the added value of fine-mapping the MHC to pinpoint genetic risk factors for autoimmune disease has been well demonstrated by numerous studies. The associations that have been found in both European and Asian populations to the same amino acids by fine-mapping the MHC suggest that the same molecular mechanism is involved, despite the differences in MHC allele frequencies seen between these ethnic groups.

MHC-based imputation approaches using genotype data, along with the use of population-specific reference panels for imputing MHC alleles and amino acids, has allowed identification of the MHC variation associated with complex diseases. Although identification is challenging, genetic variation in the MHC is of critical importance for two reasons. First, it sheds light on the development of autoimmunity, given the two hypotheses discussed above (pathogen-driven evolutionary selection of protective genes or pathogens as triggers of autoimmunity), and second, it yields greater understanding of the complexity of the human immune system. This knowledge will ultimately permit the design of better prophylactic and therapeutic strategies to achieve more balanced patient–immune responses during treatment.

## Box 1. The major histocompatibility complex locus

The major histocompatibility complex (MHC) was discovered in the mouse in 1936 [128]. It covers 0.13% of the human genome [1] and shows a high degree of polymorphism and extensive patterns of linkage disequilibrium (LD), which differ among populations. The large number of MHC alleles means each individual has a nearly unique set of peptide-presenting allotypic MHC molecules, and each MHC allotype confers the ability to bind different peptides. The MHC genes are classified into five subregions from the telomeric to the centromeric end: the extended class I, class I, class III, class II, and the extended class II regions [1]. The extended MHC region contains more than 400 annotated genes and pseudogenes that extend beyond the boundaries defining the MHC.

The class I region includes the three classic human leukocyte antigen (HLA) gene loci: HLA-A, HLA-B, and HLA-C; three non-classic HLA-E, HLA-F, and HLA-G gene loci, which show limited polymorphism compared to the classic class I loci; and other related non-coding genes and pseudogenes [1]. The main function of HLA class I molecules, which are expressed in all nucleated cells, is to present non-self antigens derived from intracellular sources, such as viruses, to CD8^+^ T cells (cytotoxic T cells), which then kill the antigen-presenting cells (APCs) [129]. CD8^+^ T cells interact with the cognate peptide-MHC I complexes via their T-cell receptor (TCR) and co-receptor molecule CD8.

The class II region includes the classic gene loci HLA-DP, HLA-DQ, and HLA-DR and also the non-classic HLA-DO and HLA-DM loci [1]. The classic genes are expressed on the surface of professional APCs, which take up antigens derived from extracellular sources [130], such as bacteria or food, and present them to CD4^+^ T helper cells. This leads to the secretion of various small proteins, including cytokines, which regulate other immune cells such as macrophages or B cells. In turn, macrophages can destroy ingested microbes, and activated B cells can secrete antibodies. CD4^+^ T cells interact with the cognate peptide-MHC II complexes via their TCR and the co-receptor molecule CD4. Non-classic molecules are exposed in internal membranes in lysosomes, which help load antigenic peptides on to classic MHC class II molecules.

The class III region contains genes involved in inflammation, for example, complement cascades (C2, C4, CFB), and in cytokine production (TNF, LTA, LTB), as well as many other genes of non-immune or unknown function that may not be involved in inflammation [1].

Overall, classic MHC I and II molecules present peptides for T-cell surveillance and are, therefore, critical for priming the cellular adaptive immune responses.

## Box 2. Clinical characteristics and prevalence of autoimmune diseases in Europeans

**Rheumatoid arthritis**

Chronic inflammation of synovial joints, with a prevalence of 0.5–1%. In some individuals, rheumatoid arthritis can damage a wide variety of body systems, including the skin, eyes, lungs, heart, and blood vessels.

**Celiac disease**

Chronic inflammation of the intestine triggered by gluten peptides in the diet and leading to flattening of the mucosa. Prevalence is 0.5–2%.

**Psoriasis**

An inflammatory skin condition characterized by rapid growth and skin cell reproduction. The disease trigger is unknown. The prevalence is 0.5–1% worldwide, but it is higher (2%) in Europeans.

**Ankylosing spondylitis**

A chronic, degenerative, and inflammatory form of arthritis, primarily affecting the spine and sacroiliac joints, and eventually leading to spinal fusion. This makes the spine less flexible and can result in a hunchback posture. It has a prevalence of 0.025%.

**Systemic lupus erythematosus**

Chronic inflammation that can affect any part of the body, although it often attacks the heart, joints, skin, lungs, blood vessels, liver, kidneys, and nervous system. It has a prevalence of 0.04–0.12%.

**Type 1 diabetes**

Characterized by the destruction of pancreatic beta-cells, leading to insufficient release of insulin from the pancreas. It has a prevalence of 0.2–0.3%.

**Multiple sclerosis**

Characterized by autoimmune attack on the central nervous system, leading to demyelination of neurons, and potentially debilitating physical and mental symptoms. It has a prevalence of 0.02%.

**Graves’ disease**

An autoimmune thyroid disorder leading to the overproduction of thyroid hormones (hyperthyroidism). Graves’ disease occurs in about 0.5% of males and 3% of females [131]. It is the most common cause of hyperthyroidism in the USA, affecting about 1 in 200 people (0.5%) according to the National Institutes of Health (https://ghr.nlm.nih.gov/condition/graves-disease#statistics).

**Inflammatory bowel disease**

A group of intestinal disorders involving chronic inflammation of the digestive tract. The two most common types of inflammatory bowel disease are Crohn’s disease (CD), which is characterized by inflammation of any part of the digestive tract, and ulcerative colitis (UC), in which the inflammation is mostly localized in the large intestine. In Europe, CD has a prevalence varying from 0.00015 to 0.2%, whereas the prevalence of UC varies from 0.0024 to 0.3% [132].

**Dermatomyositis**

A rare idiopathic myopathy characterized by inflammation, primarily of the muscles and skin. It may also affect the joints, esophagus, lungs, and heart. The disease incidence ranges from 1.2 to 17 new cases per 1,000,000 inhabitants, with a prevalence between 0.005 and 0.011% [133].

## Box 3. Infectious diseases and infection-related GWAS phenotypes

**HIV infection**

Infection by the lentiviral human immunodeficiency virus (HIV; a subgroup of retrovirus) is a global public health issue. According to the World Health Organization (WHO), 36.7 million people were living with HIV at the end of 2015 (http://www.who.int/). The virus attacks human immune cells. Over time, HIV infection develops into acquired immunodeficiency syndrome (AIDS), a condition characterized in humans by progressive loss of immune function and leading to life-threatening opportunistic infections and cancers.

**Dengue shock syndrome**

Dengue shock syndrome is the most dangerous and severe complication of infection with the dengue virus. It is characterized by increased vascular permeability, together with myocardial dysfunction and dehydration. Dengue virus is a single, positive-stranded RNA virus of the *Flaviviridae* family; it is mainly transmitted by mosquitos. Dengue is found in tropical and sub-tropical climates, mostly in urban and semi-urban regions. The WHO estimates that about half of the world’s population is now at risk.

**Hepatitis B virus infection**

Hepatitis B virus (HBV) is a double-stranded DNA virus belonging to the *Heoadnaviridae* family. The virus can cause both acute and chronic infections. Chronic infection with HBV leads to serious liver disease, often progressing to liver cirrhosis and hepatocellular carcinoma [134]. The WHO estimates that about 240 million people live with chronic HBV infection worldwide, with the highest prevalence seen in Africa and Asia.

**Hepatitis C virus infection**

Hepatitis C virus (HCV) is a single-stranded RNA virus of the *Flaviviridae* family. It has the same target as HBV—the liver—and can cause both acute and chronic infections. The WHO estimates that 130–150 million people worldwide have chronic infection; many of these will go on to develop liver cirrhosis or liver cancer. The prevalence of HCV infections is highest in Africa and Central and East Asia.

**Human papillomavirus virus infection**

Human papillomavirus virus (HPV) covers a highly diverse group of DNA papillomaviruses that are common worldwide. They can infect either mucosal or cutaneous epithelia but, in most cases, can be cleared by the human immune system. If infection persists, certain high-risk mucosal types (e.g., HPV16 and HPV18) can lead to cervical cancer and other anogenital and oropharyngeal cancers.

**Leprosy**

Leprosy is a chronic infectious disease caused by *Mycobacterium leprae*; it mainly affects the skin, peripheral nerves, mucosa of the upper respiratory tract, and eyes. It is curable using multidrug therapy, which the WHO has made available free of charge to patients worldwide since 1995. The WHO reports on leprosy in 121 countries and territories, but not in Europe, and gave a prevalence of 175,554 cases at the end of 2014. Thus, leprosy remains a serious public health problem, especially in developing countries. Leprosy is classified into five distinct clinical subtypes. At one end of the spectrum, tuberculoid leprosy (TT) is characterized by fewer lesions and resistance to mycobacteria proliferation, caused by a robust Th1 antigen-specific cellular response. In contrast, at the other end of the spectrum, lepromatous leprosy (LL) is characterized by numerous lesions and proliferation of mycobacteria because of a weak or absent cellular immune response and a dominant Th2 response. Between TT and LL, there is a range of intermediate forms and manifestations [135].

**Tuberculosis**

Tuberculosis is caused by *Mycobacterium tuberculosis*; it most often affects the lungs but can affect other parts of the body. It is one of the top infectious killers worldwide, with over 95% of cases and deaths occurring in developing countries. Sub-Saharan Africa has the highest disease prevalence. Pulmonary tuberculosis is the only transmissible form of the disease and the most common form seen in all ages (WHO, Global Tuberculosis Report 2016; http://apps.who.int/iris/bitstream/10665/250441/1/9789241565394-eng.pdf?ua=1). In 2015, the WHO estimated 10.4 million new (incident) tuberculosis cases worldwide.

**Leishmaniasis**

Leishmaniasis is caused by the protozoan *Leishmania* parasites, which are transmitted to humans by infected female sandfly bites. The disease has three forms: visceral (also known as kala-azar, the most serious form of the disease), cutaneous (the most common), and mucocutaneous. It is classified as a neglected tropical disease, and the WHO estimates that there are 900,000 to 1.3 million new cases and 20,000 to 30,000 deaths annually. Visceral leishmaniasis is endemic in the Indian subcontinent and in East Africa, while cutaneous leishmaniasis is most common in the Americas, the Mediterranean basin, the Middle East, and Central Asia. Cases of mucocutaneous leishmaniasis occur in South America (Bolivia, Peru, and Brazil; WHO Fact Sheet, updated September 2016; (http://www.who.int/mediacentre/factsheets/fs375/en/).

## Additional files

Additional file 1: Imputed MHC classic alleles and amino acids for infectious diseases. (XLSX 40 kb)

Additional file 2: GWAS SNPs for autoimmune diseases. (XLSX 61 kb)

## Abbreviations

APC: Antigen-presenting cell
AS: Ankylosing spondylitis
CD: Crohn’s disease
CeD: Celiac disease
DM: Dermatomyositis
GWAS: Genome-wide association study
HBV: Hepatitis B virus
HCV: Hepatitis C virus
HIV: Human immunodeficiency virus
HLA: Human leukocyte antigen
HPV: Human papilloma virus
IBD: Inflammatory bowel disease
KIR: Killer immunoglobulin receptor
LD: Linkage disequilibrium
MHC: Major histocompatibility complex
MS: Multiple sclerosis
NGS: Next-generation sequencing
PRG: Population reference graph
RA: Rheumatoid arthritis
SLE: Systemic lupus erythematosus
SNP: Single nucleotide polymorphism
TCR: T-cell receptor
UC: Ulcerative colitis
